# Impact of Tumour Epithelial Subtype on Circulating microRNAs in Breast Cancer Patients

**DOI:** 10.1371/journal.pone.0090605

**Published:** 2014-03-13

**Authors:** Peadar S. Waters, Roisin M. Dwyer, Cathy Brougham, Claire L. Glynn, Deirdre Wall, Peter Hyland, Maria Duignan, Mark McLoughlin, John Newell, Michael J. Kerin

**Affiliations:** 1 Discipline of Surgery, School of Medicine, National University of Ireland Galway, Galway, Ireland; 2 HRB Clinical Research Facility and School of Mathematics, Statistics and Applied Mathematics, National University of Galway, Galway, Ireland; Deutsches Krebsforschungszentrum, Germany

## Abstract

While a range of miRNAs have been shown to be dysregulated in the circulation of patients with breast cancer, little is known about the relationship between circulating levels and tumour characteristics. The aim of this study was to analyse alterations in circulating miRNA expression during tumour progression in a murine model of breast cancer, and to detemine the clinical relevance of identified miRNAs at both tissue and circulating level in patient samples. Athymic nude mice received a subcutaneous or mammary fat pad injection of MDA-MB-231 cells. Blood sampling was performed at weeks 1, 3 and 6 following tumour induction, and microRNA extracted. MicroRNA microArray analysis was performed comparing samples harvested at week 1 to those collected at week 6 from the same animals. Significantly altered miRNAs were validated across all murine samples by RQ-PCR (n = 45). Three miRNAs of interest were then quantified in the circulation(n = 166) and tissue (n = 100) of breast cancer patients and healthy control individuals. MicroArray-based analysis of murine blood samples revealed levels of 77 circulating microRNAs to be changed during disease progression, with 44 demonstrating changes >2-fold. Validation across all samples revealed miR-138 to be significantly elevated in the circulation of animals during disease development, with miR-191 and miR-106a levels significantly decreased. Analysis of patient tissue and blood samples revealed miR-138 to be significantly up-regulated in the circulation of patients with breast cancer, with no change observed in the tissue setting. While not significantly changed overall in breast cancer patients compared to controls, circulating miR-106a and miR-191 were significantly decreased in patients with basal breast cancer. In tissue, both miRNAs were significantly elevated in breast cancer compared to normal breast tissue. The data demonstrates an impact of tumour epithelial subtype on circulating levels of miRNAs, and highlights divergent miRNA profiles between tissue and blood samples from breast cancer patients.

## Introduction

Interest in microRNAs as potential non-invasive biomarkers of disease continues to grow as a result of their presence in the circulation. These short, non-coding nucleotide sequences play a pivotal role in gene regulation within the cell through translational repression of target mRNAs [1]. Evidence of their dysregulation in the circulation of patients with various cancer types has stimulated intensive investigation into their potential for detection of disease development or recurrence, determination of patient prognosis, and indication or prediction of reponse to therapy [2], [3]. It remains unclear whether circulating miRNAs originate from the diseased tissue itself, or as a result of host response to disease.

Breast cancer is a heterogeneous disease, and although mortality rates are decreasing, incidence continues to rise [4]. Stage of disease at diagnosis is directly related to outcome so early detection is key. Breast cancer is known to comprise of several distinct molecular subtypes, with subtype of disease also having a major impact on outcome [5]. A number of studies have also found miRNAs to be differentially expressed between molecular tumour subtypes [6], [7], [8]. However the relationship between circulating and tissue miRNAs, and the impact, if any, of tumour subtype on circulating miRNAs requires further elucidation.

Despite significant progress in breast cancer research, a reliable biomarker with appropriate sensitivity and specificity for detection of early disease has yet to be identified. Mammography remains the gold standard for breast cancer screening. To date it has been reported that screening mammography has a sensitivity ranging from 62.9%–87% [9], [10], [11]. The use of CA15.3 as an adjunct in screening and prognostication is also limited since it is only raised in 10% of stage I and 20% stage II breast cancers [12], [13]. Therefore the need for a specific, sensitive and non-invasive biomarker for the detection of disease, or to indicate recurrence or response to therapy is critical [14].

This group recently published a study investigating the use of a murine model of breast cancer for analysis of circulating miRNAs previously implicated in breast cancer [15]. The aim of this study was to use microArrays to analyse alterations in circulating miRNA expression during tumour progression to potentially identify novel miRNAs associated with disease. Following validation in murine samples, identified targets were then applied to blood and tissue samples from breast cancer patients and healthy control individuals to determine their true relevance in the clinical setting. Any relationship between the circulating miRNAs of interest and tumour epithelial subype was also investigated.

## Materials and Methods

### Cell Culture

The breast cancer cell line MDA-MB-231 was obtained from the American Type Culture Collection (ATCC), and cultured in Leibowitz-15 (L-15), supplemented with 10% fetal bovine serum (FBS), and 100 IU/ml and Penicillin G (2 units/mL)/Streptomycin sulphate (100 mg/mL).

### In Vivo Model

All animal experiments were licensed, and ethical approval was received from the National University of Ireland Galway Animal Care Research Ethics Committee. Similar to previous experiments [15], fifteen female athymic nude mice (Harlan Sprague-Dawley, Indianapolis, IN) received a mammary fat pad (MFP, n = 8) or subcutaneous right flank (SC, n = 7) injection of 2×10^5^ or 4×10^5^ respectively of MDA-MB-231 cells in 0.2 ml 50% Matrigel medium. Blood sampling was carried out on all animals from the lateral tail vein at weeks 1, 3 and 6 following induction (total n = 45). Blood was stored at 4°C in 2 ml EDTA tubes until required. At week 6 all mice were sacrificed by CO_2_ inhalation.

### Patient Samples

Written informed consent was obtained from each patient prior to sample collection, and the study was approved by the ethics review board of Galway University Hospital, and cpmplied with the principles of the Declaration of Helsinki. All blood samples (total n = 166: n = 83 pre-operative breast cancer, n = 83 healthy controls, Table 1) were stored in 10 ml Vacuette EDTA tubes (Grenier Bio-one) at 4°C. Breast tissue specimens (total n = 100: n = 60 breast cancer, n = 40 reduction mammaplasty controls) were obtained from patients undergoing surgery at Galway University Hospital, Galway, Ireland. Following excision, tissue samples were preserved in RNAlater® and then stored at −80°C until RNA extraction. Clinicopathological details were retrieved on each patient and are shown in Table 1.

**Table 1:** Clinicopathological characteristics of patients from whom blood samples were harvested for analysis of circulating microRNAs.

	Breast Cancer (n = 83)	Controls (n = 83)
Mean Patient Age (yr)	57.2	57.1
**Menopausal Status**		
Pre	33	37
Post	49	45
Peri	1	1
**Histological Subtype**		
Luminal A	31	
Luminal B	11	
Her2/neu	18	
Basal	23	
**Tumour Grade**		
1	9	
2	38	
3	36	
**Tumour Stage**		
1	25	
2	46	
3	12	

### microRNA Extraction

microRNA was extracted from 50 uL of murine blood or 1 mL of human blood using a modification of the TRI Reagent BD technique (Molecular Research Centre, Inc.) as described previously [16]. Total RNA was extracted from patient tissue using the RNeasy kit (Qiagen) according to manufacturers instructions. Sample concentration and purity were assessed by NanoDrop™ 1000 spectrophotometry (Nanodrop Technologies, Wilmington, DE, USA) and also using the RNA 6000 Nano Lab Chip Series II Assay with the 2100 Bioanalyzer System (Agilent Technologies, Palo Alto, CA, USA).

### MicroRNA microArray

TaqMan Low Density microRNA Array Card A v2 (Applied Biosystems) were employed, consisting of 384-well microfluidics cards targeting well-characterised miRNAs with U6 included as an endogenous control. Five animals (three animals with MFP tumours and two with SC tumours), with corresponding blood samples at week 1 and week 6 were included in the study (10 murine samples in total). A total of ten microarrays were performed, with whole blood sampled one week following tumour induction and again at 6 weeks in the presence of progressive disease. During internal validation the change in microRNA profile was then analysed across all murine blood samples at week 1, week 3 and week 6, with the “early” sample providing the baseline measurements for each animal (n = 45). Assays were run on the 7900HT Fast Real-Time polymerase chain reaction (PCR) machine(Applied Biosystems). MiRNAs were ranked according to their degree of dysregulation in relative fold change. After validation across all murine samples (n = 45), three miRNAs of interest were translated into a breast cancer patient cohort and circulating (n = 166) and tissue (n = 100) levels quantified. MicroArray-based analysis of blood samples resulted in detection of a selection of microRNAs, and the entire data set is deposited in the Gene Expression Omnibus(GEO) archive : GEO accession no. GSE47455.

### Analysis of microRNA Expression

Real-Time Quantitative Polymerase Chain Reaction (RQ-PCR) quantification of miRNA expression was performed using TaqMan® MicroRNA Assays (Applied Biosystems, Foster City, CA, USA) according to the manufacturer’s protocol. MiRNA (100 ng) was reverse-transcribed using the MultiScribe™-based High-Capacity cDNA Archive kit (Applied Biosystems). RT-negative controls were included in each batch of reactions. PCR reactions were carried out in final volumes of 10 µl using on a 7900 HT Fast Real-Time PCR System (Applied Biosystems). Briefly, reactions consisted of 0.7 µl cDNA, 1×TaqMan® Universal PCR Master Mix, 0.2 µM TaqMan® primer–probe mix (Applied Biosystems). Reactions were initiated with 10-minute incubation at 95°C followed by 40 cycles of 95°C for 15 seconds and 60°C for 60 seconds. Expression of miR-138, miR-106a and miR-191 was examined in patient samples. U6 and let-7a were used as endogenous controls to standardize miRNA expression for blood and tissue respectively [17]. Triplicate samples, validated endogenous controls, and interassay controls were used throughout. MiRNA expression levels were calculated and the threshold standard deviation for intra-assay and inter-assay replicates was <0.3. The relative quantity of miRNA expression was calculated using the comparative cycle threshold (ΔΔCt) method [18], and the lowest expressed sample was used as a calibrator.

### Statistical Analysis

Due to the magnitude and range of relative miRNA expression levels observed, results data were log transformed for analysis. Data are presented as mean+/−SD. There was no evidence against normality for the log transformed data as confirmed using the Kolmogorov-Smirnov test. The 2-sample t test was used for all 2 sample comparisons and ANOVA, followed by Tukey HSD post hoc test for inter-sample comparisons. All tests were 2-tailed and results with a *P*<0.05 were considered statistically significant. Analysis was performed in Minitab® Statistical Software (version 16.0; PA, USA) for windows.

### Ethical Standards

All animal experiments were licensed, and ethical approval was received from the National University of Ireland Galway Animal Care Research Ethics Committee. Written informed consent was obtained from each patient prior to sample collection, and the study was approved by the ethics review board of Galway University Hospital, and cpmplied with the principles of the Declaration of Helsinki.

## Results

### MicroRNA microArray

When samples from individual animals taken one or six weeks following tumour induction were compared, levels of 77 circulating microRNAs were found to be changed during disease progression, with the ten miRNAs demonstrating greatest change summarised in Table 2. Analysis was further performed to focus on targets with changes >2-fold, that were common to 4 animals (n = 44, Table 3).

**Table 2:** Top ten microRNAs demonstrating greatest changes in circulating levels in animals from week 1 to week 6 during tumour progression.

Target
miR-138
miR-128a
miR-323-3p
miR-574-3p
miR-106a
miR-191
miR-202
miR-744
miR-486
miR-130a

**Table 3:** Selection of 44 microRNAs found to be commonly dysregulated in the circulation of 4/5 animals as tumours progressed.

hsa-let-7b-002619	hsa-miR-126-002228
hsa-let-7d-002283	hsa-miR-130a-000454
hsa-let-7e-002406	hsa-miR-130b-000456
hsa-let-7g-002282	hsa-miR-140-3p-002234
hsa-miR-15b-000390	hsa-miR-142-3p-000464
hsa-miR-16-000391	hsa-miR-146b-001097
hsa-miR-17-002308	hsa-miR-186-002285
hsa-miR-19a-000395	hsa-miR-191-002299
hsa-miR-19b-000396	hsa-miR-195-000494
hsa-miR-20a-000580	hsa-miR-92a-000432
hsa-miR-21-000397	hsa-miR-92a-000432
hsa-miR-24-000402	hsa-miR-92a-000432
hsa-miR-25-000403	hsa-miR-106a-002170
hsa-miR-26a-000405	hsa-miR-106b-000443
hsa-miR-26b-000407	hsa-miR-126-002229
hsa-miR-29a-002112	hsa-miR-130a-000455
hsa-miR-30b-000602	hsa-miR-130b-000457
hsa-miR-30c-000419	hsa-miR-140-3p-002235
hsa-miR-31-002279	hsa-miR-142-3p-000465
hsa-miR-92a-000431	hsa-miR-146b-001098
hsa-miR-106a-002169	hsa-miR-186-002286
hsa-miR-106b-000442	hsa-miR-191-002300

### Validation of microArray Data in Murine Samples

**Figure 1:**
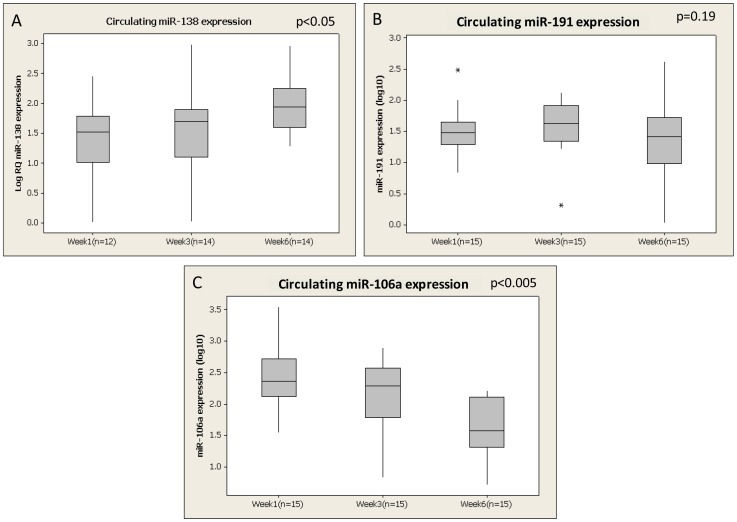
Validation of changes in circulating levels of microRNAs identified using microArray analysis in murine blood samples at week1, week 3 and week 6 following tumour induction (A) Circulating miR-138 (B) Circulating miR-191 (C) Circulating miR-106a. * denotes outliers.

miR-138, miR-191 and miR-106a were then selected and further validated across all murine blood samples (total n = 45: n = 15 week 1, n = 15 week 3, n = 15 week 6). In agreement with the microArray data, analysis of all murine samples (n = 45) revealed miR-138 to be significantly elevated in the circulation of animals during disease development (p<0.05, Figure 1A). Although found to be altered on microArray, no significant change in circulating miR-191 during disease progression was observed (p = 0.19, Figure 1B). However, when investigated in the blood of mice bearing subcutaneous tumours, miR-191 was significantly downregulated over time (p<0.05, results not shown). Further analysis of miR-106a revealed a significant decrease from week 1 (Mean ± SEM; 2.39±0.08 log_10_ Relative Quantity (RQ)) to week 6 (1.61±0.1 log_10_ RQ, Figure 1C).

### Translation Into Patient Samples - Circulating and Tissue Levels of microRNAs

**Figure 2:**
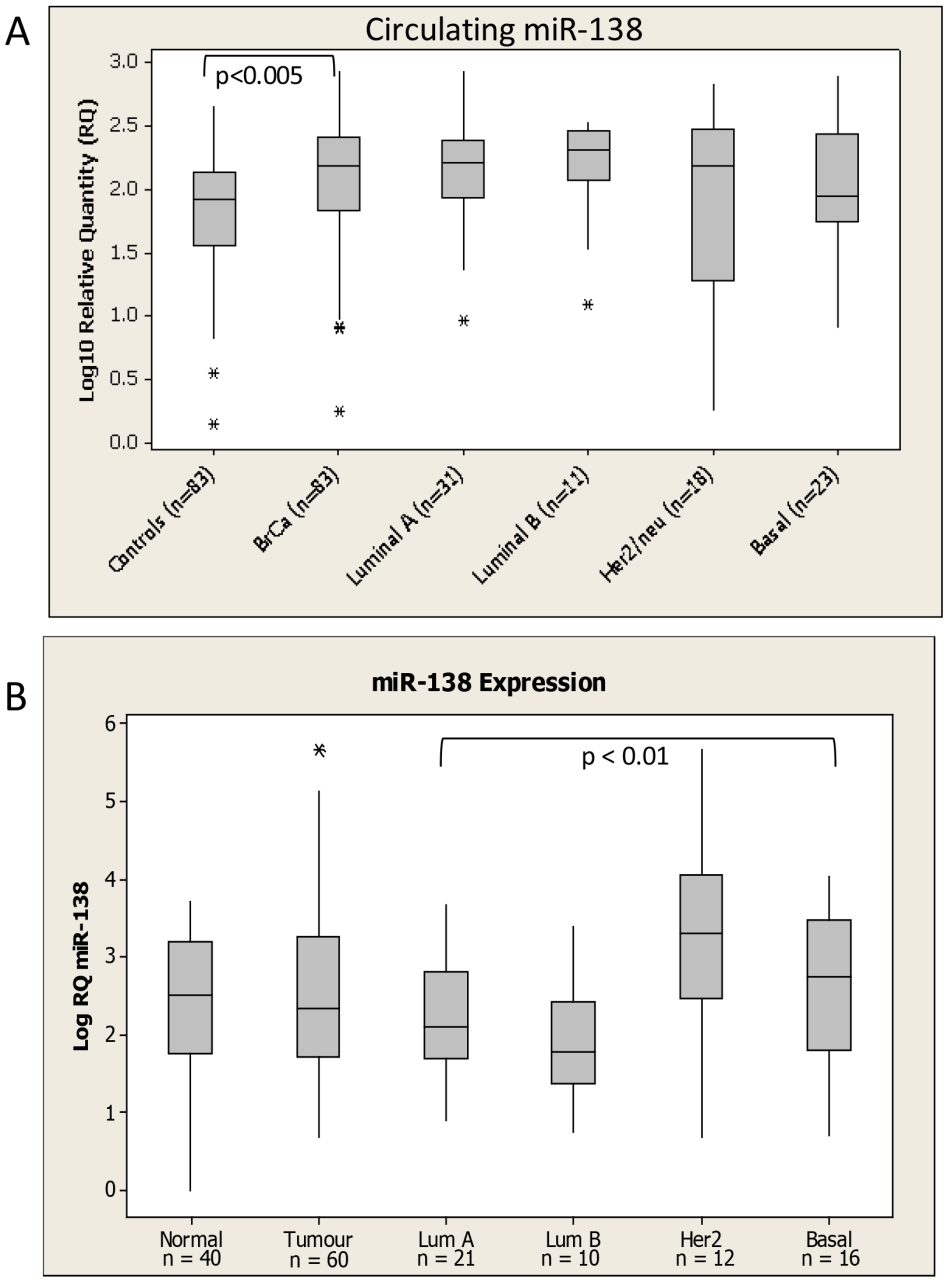
miR-138 levels in (A) Circulation and (B) tissue of Breast Cancer Patients and healthy controls. Levels in breast cancer patients were further subdivided based on epithelial subtype of the disease. * denotes outliers.

miR-138, miR-191, and miR-106a were detectable in the circulation of all breast cancer patients and healthy controls included in the study (n = 166). miR-138 was found to be significantly up-regulated in the circulation of patients with breast cancer (2.05±0.06 log_10_ RQ) compared to healthy controls (1.83±0.05, log_10_RQ, p<0.005, Figure 2A). No relationship with tumour epithelial subtype was observed. The levels of this miRNA were also investigated in tumour tissue(n = 60) and healthy tissue harvested at reduction mammoplasty(n = 40). No significant dysregulation was observed with miR-138 expression when comparing tumour to normal tissue. Interestingly, however, miR-138 was significantly dysregulated across breast cancer subtypes (ANOVA p<0.01), with higher levels detected in the HER2 and basal subtype tissues (Figure 2B).

**Figure 3:**
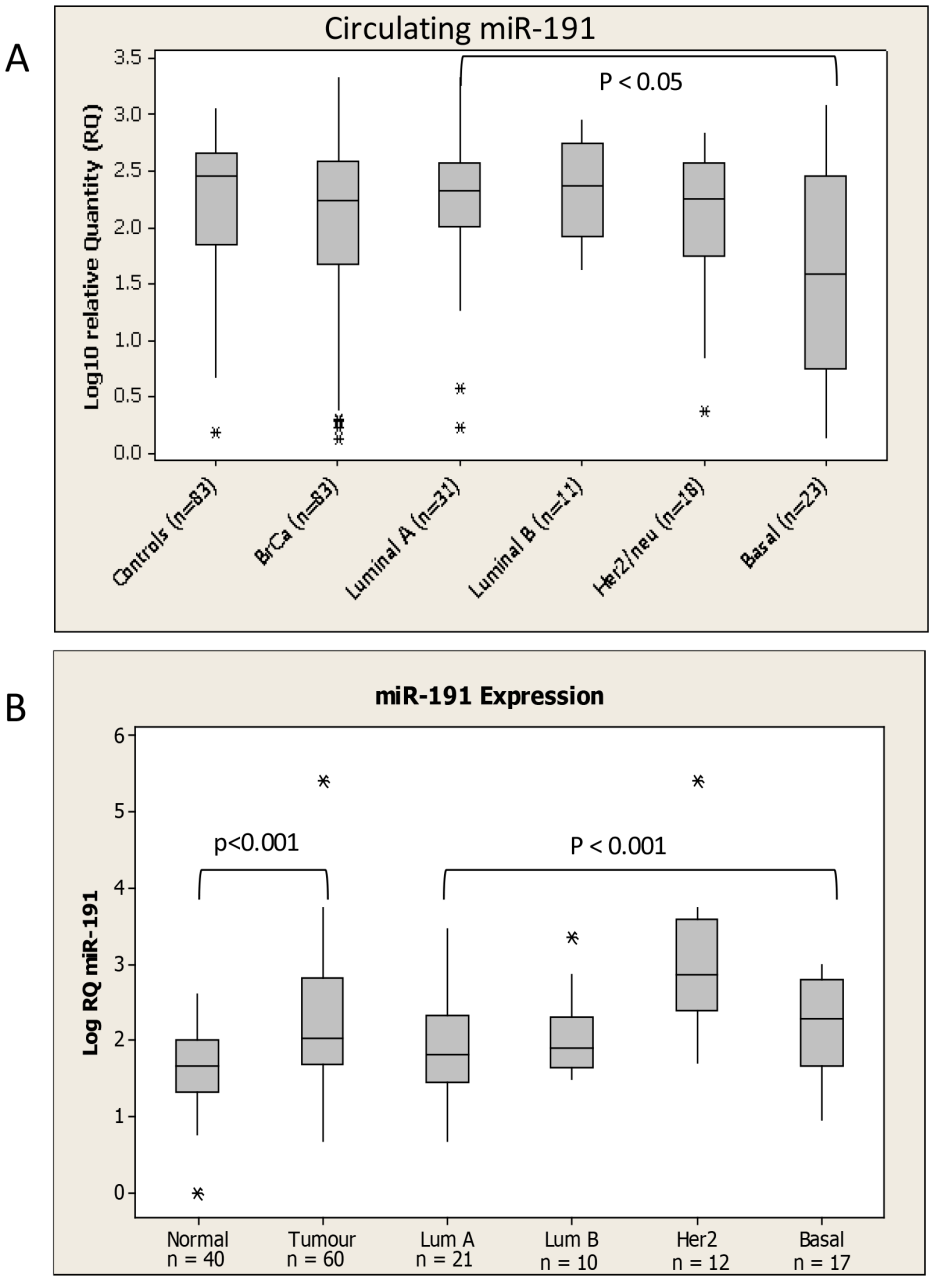
miR-191 levels in (A) Circulation and (B) tissue of Breast Cancer Patients and healthy controls. Levels in breast cancer patients were further subdivided based on epithelial subtype of the disease. * denotes outliers.

miR-191 was not significantly altered overall in breast cancer patients compared to controls (Figure 3A). However, circulating levels of the microRNA were significantly dysregulated across epithelial subtype (ANOVA p<0.05), with the lowest levels observed in patients with Basal breast cancer (Figure 3A). In contrast, tissue levels of miR-191 were significantly higher in breast cancer patients compared to healthy controls (p<0.001, Figure 3B). Further, expression of miR-191 was significantly altered across epithelial subtype(p<0.001), with higher levels detected in the Her2 and Basal subtypes.

**Figure 4:**
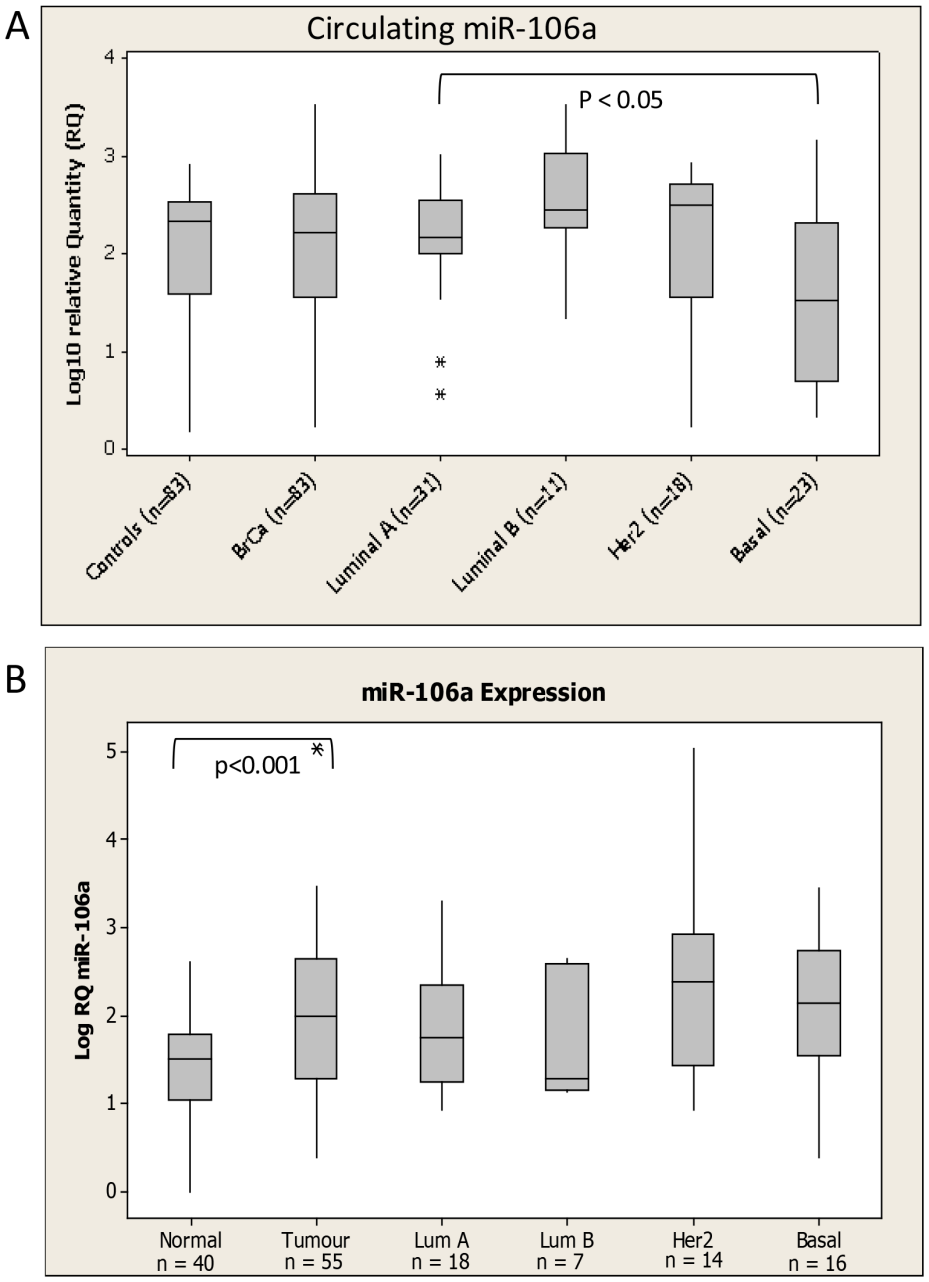
miR-106a levels in (A) Circulation and (B) tissue of Breast Cancer Patients and healthy controls. Levels in breast cancer patients were further subdivided based on epithelial subtype of the disease. * denotes outliers.

Similarily, circulating miR-106a was not significantly altered overall in breast cancer patients compared to controls (Figure 4A). However, as observed with miR-191, miR-106a displayed differential expression across epithelial subtype (ANOVA p<0.05). Indeed, when evaluated in isolation, both miR-191 and miR-106a were significantly down-regulated in patients with basal breast cancer compared to healthy controls (p<0.05, p<0.005 respectively). miR-106a was also found to be significantly elevated in breast tumour compared to healthy breast tissue (p<0.001, Figure 4B). No significant correlation to patient clinicopathological characteristics such as tumour stage, grade or menopausal status was observed.

## Discussion

MicroRNAs have tremendous potential as circulating biomarkers of diesase, however challenges remain to their successful application in the clinical setting. In a heterogeneous disease such as breast cancer, the impact of tumour characteristics on circulating miRNAs, and indeed the relationship between circulating and tissue levels, require further investigation. The current study describes analysis of circulating miRNAs in an animal model of breast cancer over 6 weeks of tumour progression. A selection of dysregulated miRNAs were then validated within the murine samples and targets of interest further investigated in tissue and blood samples harvested from breast cancer patients and healthy control individuals.

miR-138, analysis of which has not previously been reported in patient samples, was identified as a potential circulating marker of breast cancer, with levels significantly elevated over time as tumours progressed in animals. This held true in the larger validation cohort and further, levels were found to be significantly elevated in the circulation of breast cancer patients compared to healthy controls. This data hightlights the validity of the murine model used to identify potentially important circulating miRNAs that are translatable to the patient setting. Indeed it may be interesting to compare circulating levels in mice bearing tumours originating from varying molecular subtypes of breast cancer. Although there are inherent flaws to any model system, a major benefit is that other comorbidities or confounding factors in patients that may impact circulating miRNAs are absent from the animal model.

Although miR-138 has not previously been analysed in breast tissue samples, on a cellular level it has been implicated as a tumour suppressor [19], [20], [21], potentially through targeting Neutrophil gelatinase-associated lipocalcin(NGAL) [19] or hTERT [21]. In the current study, on a tissue level, miR-138 expression was not significantly changed in breast tumour samples compared to healthy controls, but was found to be significanlty altered across epithelial subtype, with the highest levels detected in Her2 amplified samples.

Both miR-191(in subcutaneous model) and miR-106a were found to decrease at a circulating level in the murine model, but in patient blood samples there was no significant difference between levels in breast cancer patients and healthy controls. Considering the murine model used was based on injection of the basal breast cancer cell line MDA-MB-231, it was interesting to find that in both cases circulating levels were signfiicantly decreased in patients with basal breast cancer compared to healthy controls. It is noteworthy that at a tissue level, miR-191 was previously reported to have lower expression in ER negative tumours compared to ER positive [22]. It has also been known to fuction as a tumour supressor through regulation of CDK6 in thyroid cancer [23]. MiR-106a has also been associated with basal breast cancer in the tissue context, although levels were reported to be elevated rather than decreased supporting its role as an oncomiR [7], [24]. Reported target genes of miR-106a include RUNX3, resulting in multidrug resistance in gastric cancer [25]. Hu et al [26] previously analysed miR-191 levels in serum of breast cancer patients and healthy controls, and similar to the current findings reported no significant difference, and further suggested use of miR-191 as an endogenous control. However in that study all samples were pooled and so not stratified based on tumour characteristics [26].

The data presented here highlights divergent miRNA profiles observed between tissue and blood samples from breast cancer patients, and support the idea that selected miRNAs may play a more important role in the tumour microenvironment than in the circulation. Further understanding of the factors contributing to circulating levels of miRNAs, and the impact of tumour, and other patient characteristics, on the miRNA profile is key to their successful implementation as biomarkers of disease.

A number of studies have previously reported elevation of miR-191 in tissue of a variety of cancer types including lung [27], hepatocellular [28] and breast [22], [29]. Similarily, miR-106a has a well chacterized asoociation with cancer, and has been reported to be upregulated in breast cancer compared to control tissue [30]. While not elevated at a circulating level, the current study also found signficantly elevated levels of miR-191 and miR-106a in breast cancer compared to normal breast tissue harvested at reduction mammaplasty.

## Conclusions

Fluctuations in miRNA levels during tumour development, and determination of whether particular miRNAs could be indicative of tumour subtype or aid selection of optimal treatment strategies will be key to successful implementation in the clinical setting. MiRNAs that are detectable at the early stages of tumourigenesis have the potential to be used as a screening tool alongside CA 15.3 and mammography to improve early breast cancer detection. Identification of the optimal panel of miRNAs for disease diagnosis, prediction of prognosis, or response to therapy will be dependent upon progress in this area.
